# Clinicopathological and molecular characteristics of second primary colorectal cancer in cancer survivors including MSI-H status and anatomical distribution

**DOI:** 10.3389/fonc.2026.1952368

**Published:** 2026-10-23

**Authors:** Yadong Liu, Xuejuan Duan, Shiwei Yang, Yayu Zhang, Shaohui Han

**Affiliations:** 1 Department of Oncology, Hebei General Hospital, Shijiazhuang, China; 2 Department of Radiation Oncology, The Fourth Affiliated Hospital of Hebei Medical University, Shijiazhuang, China; 3 Department of Gland Surgery, Hebei General Hospital, Shijiazhuang, China; 4 Department of General Practice, Hebei General Hospital, Shijiazhuang, China; 5 Department of Thoracic Surgery, Hebei General Hospital, Shijiazhuang, China

**Keywords:** colorectal cancer, microsatellite instability, MMR, MSI-H, second primary cancer

## Abstract

Accurate data on microsatellite instability (MSI) in second primary colorectal cancer (CRC) are needed for precision oncology and guideline development. This study includes structured clinicopathological records from 77 cancer survivors diagnosed with second primary CRC. Variables encompass demographics, prior malignancy and treatment history, tumor location, histology, MSI status assessed via immunohistochemistry and polymerase chain reaction (PCR), and timing between malignancies. Data were systematically extracted following clear inclusion and exclusion criteria, with standardized definitions and quality checks. MSI status was determined using staining for four mismatch repair proteins, with PCR confirmation for equivocal results. The dataset’s structure ensures accessibility, interoperability, and reproducibility. It enables analyses of MSI prevalence, anatomical predictors, and clinical associations in second primary CRC, supporting secondary research on risk factors, biomarker correlates, and population-level outcomes.

**FAIR^2^ CERTIFIED™** Explore interactive data here: 10.71728/senscience.qs2f-h81p

## 1 Introduction

Second primary malignancies represent an increasing clinical consideration as cancer survival rates improve, creating a growing population at risk for subsequent neoplasms (1–6). Second primary colorectal cancer (CRC) is among the most common of these malignancies, requiring comprehensive characterization of its clinicopathological features to inform surveillance strategies, diagnostic workup, and therapeutic interventions. Microsatellite instability-high (MSI-H) status is a key molecular phenotype in CRC (7–10), relevant to both precision medicine protocols and fundamental research in tumor biology, immunogenicity, and response to immune checkpoint inhibition (11, 12). However, data resources explicitly describing the features and determinants of MSI-H in second primary CRC among cancer survivors are limited, hindering robust analytics, meta-research, and artificial intelligence (AI)-driven approaches.

The dataset described herein provides a systematically curated record of 77 consecutive cancer survivors diagnosed with second primary CRC, compiled from a single institutional cohort. Each record integrates variables capturing demographic information, comorbidities, original primary and subsequent cancer diagnoses, temporal relationships between events, anatomical localization, pathologic subtyping, molecular phenotype [specifically MSI-H status as assessed by immunohistochemistry (IHC) and polymerase chain reaction (PCR) where indicated], and relevant exposures such as prior systemic chemotherapy (13). Additional clinical descriptors include smoking history, the presence or absence of synchronous versus metachronous presentation, and documentation of distant metastasis at second primary CRC diagnosis (14). Variables are defined according to standard biomedical ontologies and harmonized for interoperability with current clinical research data models.

Data capture was designed to support reproducibility and transparent traceability of source material. Inclusion and exclusion criteria ensured the dataset comprises only cases with confirmed distinct primary tumors, validated by histopathology, and complete documentation of MSI/MMR status. Universal reflex mismatch repair (MMR) IHC was applied to all newly diagnosed CRC specimens according to institutional policy, and equivocal or discordant results were resolved by prespecified molecular protocols, minimizing missingness and maximizing comparability between cases. Clinical, pathological, and molecular data were extracted from electronic medical records and verified through independent review, with structured coding for all key fields.

The resulting tabular dataset consists of 77 rows (one per patient) and 20 columns (variables), representing a comprehensive matrix suitable for cross-sectional and multivariate analyses. Statistical summaries of each variable are provided to facilitate immediate characterization and benchmarking. All variables are defined with units, value constraints, and controlled vocabularies where applicable; binary phenotypes and exposures are recorded as integer flags. Categorical descriptors—such as cancer type, anatomical subsite, and histological subtype—are standardized to support data integration in meta-analyses or federated learning contexts.

This data article documents the dataset’s structure, provenance, and variables in alignment with the FAIR² principles, prioritizing findability, accessibility, interoperability, and reusability for diverse analytical workflows. Machine-actionable metadata are provided for each variable, ensuring compatibility with AI and machine learning (ML) pipelines, and supporting workflows for both hypothesis-driven and agnostic discovery science. Special attention is given to provenance, method transparency, and the explicit recording of potential sources of bias, such as selection and ascertainment artifacts, to enable critical evaluation and responsible secondary use.

By supplying detailed definitions, code lists, and context-rich variable documentation, this publication aims to lower barriers to the ethical and transparent reuse of clinicopathological data on second primary CRC. The resource is intended to support data-driven research into cancer epidemiology, biomarker validation, treatment outcomes, and the development of explainable and responsible AI in oncology. The dataset is available for reanalysis subject to institutional data sharing policies and can be extended or linked to larger multi-institutional resources, contributing to reproducible evidence synthesis and translational research in the field of secondary colorectal malignancies.

## 2 Methods summary

### 2.1 Study design

This investigation was structured as a retrospective observational study with the explicit aim of characterizing the clinicopathological attributes, anatomical predictors, and prevalence of MSI-H in cases of second primary CRC among individuals with previous cancer diagnoses. The study cohort included all eligible consecutive cases identified within a hospital-based oncology follow-up program, restricted to diagnoses made between May 2019 and March 2025. Rigorous eligibility criteria were systematically enforced to ensure analytical consistency: inclusion required documented histopathological confirmation of a previous primary malignancy, a subsequent diagnosis of second primary CRC substantiated by endoscopic and pathological review, and the availability of complete clinicopathological documentation for the second primary CRC, including definitive MSI and MMR status. Exclusion criteria comprised known hereditary CRC syndromes at the time of initial diagnosis, irretrievable missing or ambiguous data, and inability to distinguish the CRC as a discrete primary tumor. Institutional review board approval was obtained (Ethics Approval No. 2025-339), with formal waiver of individual consent in light of the retrospective non-interventional design. The study adhered to ethical standards and methodological transparency as outlined by the Declaration of Helsinki and the STROBE (Strengthening the Reporting of Observational Studies in Epidemiology) reporting guidelines. Upon the systematic application of these criteria, the final analytic sample encompassed 77 consecutive survivors meeting all protocol requirements. Use of a universal reflex MMR IHC protocol ensured uniform ascertainment of MSI/MMR status, and cases failing completeness or violating eligibility were methodically excluded. MSI status was inferred from MMR IHC, and that PCR-based MSI testing was used only for cases with equivocal IHC.

During the study period, 82 patients with a second primary CRC after a previous malignancy were identified. Of these, one was excluded because of a known hereditary CRC syndrome, three were excluded because of irretrievable missing or ambiguous data (including two with unresolved MSI/MMR status), and one was excluded because the CRC could not be clearly identified as a separate primary tumor. The remaining 77 patients formed the final dataset.

### 2.2 Data collection

All primary variables relevant to clinical, demographic, and treatment history were extracted through a standardized workflow from institutional oncology databases and patient medical records. For cohort assembly, eligible cases were first identified within the hospital’s oncological registry and then verified against electronic health records to confirm accuracy and completeness of reported clinical events, covering both primary and subsequent cancer episodes. Data extraction followed a prespecified, structured protocol to ensure comparability and traceability; each variable—such as age at each cancer diagnosis, sex, comorbidities (including hypertension, diabetes mellitus, and hyperlipidemia), smoking history, types of first and second cancers, prior chemotherapy, interval between diagnoses, timing of presentation, anatomical location of CRC, histopathological subtype, metastatic status, and MSI-H/MMR status—was precisely defined according to an operational data dictionary. All field-level coding definitions and contextual clarifications were applied prior to abstraction to minimize interpretive ambiguity. Before final dataset assembly, extracted records underwent thorough assessment for missing data, inconsistencies, or ambiguous entries. Any uncertainty was resolved through re-examination of the original patient files, and records missing unresolvable MSI/MMR data were excluded in accordance with protocol. Only datasets with fully documented variables were included in subsequent analyses, thereby supporting analytic fidelity.

### 2.3 Assessment of MSI and MMR status

The evaluation of MSI and MMR status followed a multi-step laboratory protocol to maximize diagnostic accuracy and reproducibility. Tumor specimens from the second primary CRC were initially formalin-fixed and paraffin-embedded, with serial 4-μm sections prepared for downstream analysis. IHC was performed automatically for the four key MMR proteins (MLH1, MSH2, MSH6, and PMS2) using validated antibody panels. Each staining procedure incorporated internal positive controls—non-neoplastic crypt epithelium, intratumoral lymphocytes, and stromal cells—to confirm technical adequacy; slides were considered evaluable only if these controls displayed unambiguous nuclear staining. Experienced pathologists interpreted the results according to standardized criteria: detectable nuclear staining in tumor cells for any MMR protein indicated retained expression, whereas the complete absence of staining in the presence of positive internal controls was interpreted as loss. Heterogeneous or focal staining patterns were classified as retained but flagged for independent secondary review. Cases with equivocal or suboptimal technical results were subjected to repeat sectioning and restaining, and all unresolved or ambiguous findings underwent independent evaluation by a second gastrointestinal pathologist. Reclassification required consensus. If IHC remained insufficient for definitively resolving MMR status, PCR-based MSI assays were implemented, targeting a pentaplex panel of mononucleotide markers (BAT-25, BAT-26, NR-21, NR-24, and NR-27) on the original tumor samples. The presence of instability at two or more markers defined MSI-high status. Within this workflow, the term “dMMR” was used for immunohistochemically detected MMR deficiency, and “MSI-H” reflected molecular instability on PCR; cases may be reported as MSI-H/dMMR where both were observed.

### 2.4 Statistical analysis

To optimize the interpretability and reproducibility of cohort characterizations, as well as the identification of MSI-H predictors, established statistical protocols were rigorously followed. Baseline cohort attributes, including demographics and clinical features, were summarized using descriptive statistics: continuous variables were reported as medians with interquartile ranges (IQRs), while categorical variables were presented as frequencies and percentages. Univariate analyses assessed the association between MSI-H status and each individual clinicopathological variable. Two-sided Fisher exact tests were employed for categorical descriptors, and Mann–Whitney *U* tests were used for continuous data, supplemented by reporting odds ratios (ORs), 95% confidence intervals (CIs), and Cramér’s *V* statistic for effect size. For the selection of variables considered in subsequent modeling steps, a significance threshold of p<0.20 was used. All variables reaching this threshold and select clinical covariates (hypertension, gastrointestinal first primary cancer, and age at second primary diagnosis) were entered into a multivariable logistic regression model. Model fitting was performed using maximum likelihood estimation with Newton–Raphson iteration. Outputs included standardized coefficients ( β), standard errors (SEs), adjusted ORs, 95% CIs, and two-sided p-values. Multicollinearity among covariates was evaluated using the variance inflation factor (VIF), and no parameter exceeded a VIF of 3.5, indicating acceptable model stability. Because the cohort included only 16 MSI-H events, the multivariable model has a low number of events per variable, and its estimates should be regarded as exploratory. Pre-specified sensitivity analyses assessed associations between anatomical location and MSI-H status across key subgroups. These included restriction to metachronous cases (diagnostic interval >0 months), stratification by age at diagnosis of the second primary CRC ( <65 versus ≥65 years), by history of prior chemotherapy, and by type of primary cancer (gastrointestinal versus non-gastrointestinal). Fisher exact tests and recalculated ORs were used for all subgroup comparisons. All statistical analyses were performed using R version 4.3.2 and SciPy version 1.11.4. Derived results, figures, and corresponding metrics were fully integrated into the analytic dataset and documentation to support transparency and reproducibility.

### 2.5 Dataset finalization

The preparation of the final dataset adhered strictly to institutional guidelines and best practices in data quality assurance. All extracted data first underwent systematic review and cleaning to address typographical errors, inconsistent coding, and discrepancies in variable formatting. Harmonization procedures ensured full alignment of variable names, categorical definitions, and value encodings with protocol and data dictionary requirements. Continuous variables were subjected to outlier detection, with potentially anomalous or biologically implausible entries flagged for source verification. Dataset completeness and logical coherence were subsequently verified, with cross-field consistency checks such as temporal validation of cancer diagnosis intervals. Any residual discrepancies were resolved by reference to the original medical and registry records. Only cases in which all core variables—including definitive MSI-H/MMR status—were documented and cross-validated were retained for analysis. After final confirmation, the analytic dataset was exported for statistical procedures. Structured metadata was generated to accompany the archived dataset, encompassing field names, types, value schemas, and versioning details to support future reuse and reproducibility. The dataset was prepared for both immediate analysis and long-term stewardship, in accordance with institutional and publication data-sharing policies.

## 3 Data overview

### 3.1 Data summary

The dataset comprises systematically curated records from 77 cancer survivors diagnosed with second primary CRC between May 2019 and March 2025 at a single institutional follow-up program. Each case was selected according to strict inclusion and exclusion criteria, ensuring the presence of a histopathologically confirmed first primary malignancy, definitive diagnosis of second primary CRC with available clinicopathological records, and determinate MSI-H or MMR status. Cases associated with hereditary CRC syndromes, incomplete core variable data, or ambiguous tumor classification were excluded.

The dataset includes variables capturing patient demographics (sex and age at both primary diagnoses), relevant comorbidities (hypertension, diabetes mellitus, hyperlipidemia, and smoking history), oncology-specific features (type and anatomical site of both first and second primary cancers, histopathological subtype, and metastatic status), previous chemotherapy, and timing metrics (interval between cancer diagnoses and synchronous/metachronous presentation). Key clinicopathological outcome variables include second CRC location and MSI-H status, all determined through a standardized two-tier workflow of IHC and—where necessary—PCR-based MSI assays. Full traceability from data abstraction to laboratory diagnostic workflows is preserved via comprehensive metadata and field-level documentation.

Dataset harmonization ensured alignment with protocol-defined variable names, categorical encodings, and units (e.g., ages in years and intervals in months). Missing, inconsistent, or ambiguous entries were systematically resolved or excluded prior to finalization. Statistical summaries provide transparent distributions and value counts for each field, and all definitions are supported by an explicit data dictionary. The resulting tabular dataset supports queries into anatomical, temporal, and molecular features of second primary CRC in this survivor cohort. It is suitable for descriptive and exploratory analyses, hypothesis generation, and pooled or federated analyses with other cohorts. Because of its modest size, it is not intended for the stand-alone development of predictive or machine-learning models.

#### 3.1.1 Total number of records

The dataset comprises 77 records, each representing an individual cancer survivor diagnosed with a second primary CRC within the defined institutional cohort. All records contain complete entries for the full variable set, adhering to the eligibility and data completeness criteria specified by the study protocol.

#### 3.1.2 Coverage

A total of 19 distinct types of first primary malignancies are represented, including breast cancer (13 cases), lung cancer (10), colon cancer (9), gastric cancer (7), esophageal cancer (6), rectal cancer (5), prostate cancer (5), bladder cancer (5), endometrial carcinoma (4), and renal cell carcinoma (3), among others. For the second primary cancer, 50 records are classified as colon cancer and 27 are categorized as rectal cancer. Five distinct anatomical locations for the second primary CRC are present: rectal cancer (27 cases), sigmoid colon cancer (22), ascending colon cancer (13), descending colon cancer (10), and transverse colon cancer (5).

#### 3.1.3 Attribute diversity

The dataset contains 15 primary measured variables, encompassing demographic details (age at diagnosis for first and second cancers and sex), comorbidities (hypertension, diabetes mellitus, and hyperlipidemia), lifestyle factors (smoking history), cancer types (first and second cancers), anatomical site of second primary CRC, histopathological subtype, presence or absence of distant metastasis, MSI-H status, synchronous or metachronous presentation, prior chemotherapy receipt, and the time interval between diagnoses. Pathological diversity for the second primary CRC is described by three unique subtypes: adenocarcinoma (65 cases), carcinoma *in situ* (9), and mucinous adenocarcinoma (3). The sex distribution includes 48 male and 29 female patients.

#### 3.1.4 Quantitative attributes

Key quantitative attributes and their statistics include the following: age at first primary diagnosis: range, 29.5–85.0 years; mean, 63.9 years; standard deviation (SD), 11.2; age at second primary CRC diagnosis: range, 36–85 years; mean, 68.7 years; SD, 9.3; time interval between diagnoses: range, 0–359 months; mean, 60.0 months; SD, 81.9.

Binary comorbidity indicators show the following: hypertension: present in 28/77 (36.4%); diabetes mellitus: present in 16/77 (20.8%); hyperlipidemia: present in 6/77 (7.8%); smoking history: present in 35/77 (45.5%).

Clinical and pathological characteristics: previous chemotherapy administered in 46/77 (59.7%); synchronous presentation (interval = 0 months) occurred in 25/77 (32.5%); metachronous occurred in 52/77 (67.5%); distant metastasis present in 18/77 (23.4%); MSI-H status positive in 16/77 (20.8%); second primary CRC as colon cancer in 50/77 (64.9%); rectal cancer in 27/77 (35.1%).

#### 3.1.5 Data completeness

All 77 records in the released dataset are complete for every variable. This completeness results from the complete-case selection described in Section 2: patients whose missing or ambiguous data could not be resolved from the source records were excluded before the dataset was finalized. Users should therefore not assume that the source population had no missing data, and should consider possible selection bias (see Section 5.2).

### 3.2 Dataset availability

The dataset is FAIR²-certified and publicly available under the Open Data Commons Attribution License (ODC-By v1.0), permitting unrestricted reuse with appropriate attribution. Access is provided via the following: (1) the FAIR² Data Portal for visual cohort exploration, scalar metrics, and data quality dashboards; and (2) a downloadable FAIR² Data Package containing raw and processed data, structured metadata, transformation scripts, and complete methodological documentation. The dataset is archived and indexed with a persistent identifier in the SenScience ecosystem and linked to the broader FAIR² data infrastructure. All experimental procedures and data processing steps are fully described in the metadata and methods files. Access and documentation are provided at: https://sen.science/doi/10.71728/senscience.qs2f-h81p.

### 3.3 FAIR^2^ compliance introduction

The FAIR² Compliance Certification Table (**Table 1**) presents a concise summary of the dataset’s alignment with the FAIR (Findable, Accessible, Interoperable, Reusable) principles, as well as the enhanced standards of AI-Readiness and Responsible AI (FAIR²). The table evaluates key compliance domains, including metadata quality, provenance documentation, interoperability, accessibility protocols, and integrated ethical and ML readiness considerations. Each category is systematically assessed to reflect the verifiable status of policy adherence, technical implementation, and procedural robustness. Where applicable, evidence from dataset documentation, metadata records, and workflow protocols is cited to support certification claims. This structured assessment supports transparent reporting, ensures clarity regarding dataset usability for both human and machine agents, and provides a traceable record of compliance with contemporary data stewardship requirements. The table functions as an institutional record and a practical guide for data consumers seeking to assess the dataset’s suitability for secondary analyses, AI application, and responsible reuse.

**Table 1:** FAIR^2^ compliance certification of the second primary colorectal cancer dataset across the Findability, Accessibility, Interoperability, Reusability, AI-Readiness, and Responsible AI domains.

Criteria	Details	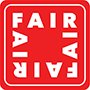
Findability (F)		
F1. Unique identifier	A persistent, institutionally managed identifier is assigned upon dataset archiving. A digital object identifier (DOI) will be registered upon publication to support citation and traceability.	
F2. Metadata	Metadata comprises structured fields describing title, authorship, institutional affiliation, cohort timeframe, eligibility criteria, variable definitions, and laboratory/analytical methods. The metadata includes explicit provenance and context for each variable. Supplementing with additional controlled vocabularies would further strengthen interoperability.	
F3. Metadata includes data identifiers	Each metadata record includes an explicit reference to the dataset’s persistent identifier, facilitating unambiguous linkage between the descriptive metadata and the actual data file.	
F4. Searchable metadata	Key variable and method terms conform to schema.org types and will be indexed using controlled vocabularies (e.g., cancer ontology and units schema). Full mapping to external vocabularies is planned for enhanced searchability.	
Indexed in repositories	The dataset is deposited in the institutional data repository and will be indexed in the FAIR² Data Portal and DataCite. Further indexing in domain-specific repositories is under consideration.	
Accessibility (A)		
A1. Open access	The dataset is accessible without restriction, in accordance with open science and transparent data-sharing policies.	
A2. Long-term access	Long-term retention is ensured through institutional archival infrastructure, with systematic backup procedures and version tracking to mitigate data loss.	
API access	Structured data portal access and download endpoints are provided; API-based retrieval and programmatic querying are supported using standard protocols.	
Interoperability (I)		
I1. Standardized formats	Data are available in open, non-proprietary CSV format. Schema definition files and metadata conform to MLCommons Croissant (v1.0) and schema.org, supporting interoperability with analytical and AI tooling.	
I2. Controlled vocabularies	Key categorical fields (e.g., cancer type and comorbidities) utilize standardized terms aligned with internal dictionaries and mapped to external ontologies where possible.	
I3. Cross-platform integration	Data structure and metadata comply with W3C PROV-O, MLCommons Croissant, and schema.org, enabling integration with health, epidemiological, and computational research workflows.	
Reusability (R)		
R1. Comprehensive and rich documentation	Extensive documentation accompanies the dataset, including detailed descriptions of study design, variable definitions, cohort selection, laboratory procedures, data validation, and a formal data dictionary.	
R1.1. License	Distributed under the Open Data Commons Attribution License (ODC-By v1.0), permitting use, modification, and sharing with required attribution to the original sources and creators.	
R1.2. Detailed provenance	Complete provenance is tracked for all data, specifying origin (clinical records, laboratory assessment, and pathologist review), version history, transformation steps, and responsible personnel at each stage.	
R1.3. Domain-relevant standards	Variable definitions, laboratory practices, and reporting align with relevant oncological and epidemiological standards, including STROBE and laboratory QA/QC protocols.	
Versioning and updates	Version control is maintained throughout dataset curation. Release notes document all updates and corrections; prior dataset iterations remain available for reference.	
AI-Readiness (AIR)		
Structured for machine learning	Variables are numerically or categorically encoded and labeled for direct ingestion into supervised and unsupervised machine learning pipelines. The structure clearly distinguishes input features and outcome variables (e.g., MSI-H status).	
Scalable	Dataset format and minimal preprocessing requirements ensure compatibility with high-performance and large-scale computational environments.	
Training and validation sets	Dataset structure supports division into training, validation, and test sets by providing consistently coded and complete records, enabling stratified or random sampling based on analytic requirements.	
Responsible AI (RAI)		
Ethical standards and misuse	The dataset is restricted to research on clinicopathological features of cancer; it is not intended for direct clinical decision-making or use outside the specified context. Documentation provides clear guidance to prevent off-label or ethically inappropriate applications.	
Biases in the dataset	Demographic, geographic, and temporal biases may be present: the cohort is drawn from a single institution and reflects the distribution of cancer types and comorbidities in that setting. No weighting or post-stratification for representativeness has been performed.	
Data privacy and security	The dataset contains no direct personal identifiers; all records are de-identified according to institutional and legal standards. Data integrity is protected via audit trails and restricted access to source files.	
Fairness and non-discrimination	Consistent inclusion/exclusion criteria minimize differential selection biases, but variability in demographic and clinical representation may affect performance and generalizability in external AI models.	
Explainability and interpretability	All variables are precisely documented with operational definitions, units, value labels, and, where applicable, references to underlying clinical or laboratory standards. Data transformations are transparently documented.	
Ethical and social impact	Dataset reuse is intended to support research on molecular and clinicopathological predictors in cancer survivors. Guidance is provided to discourage applications that may impact patient care without appropriate validation.	
Human-in-the-loop (HITL) considerations	Initial data labeling, ambiguity resolution, laboratory interpretation, and pathological review involved human experts. All manual intervention steps are documented; users are advised to validate model outputs with clinical experts in HITL applications.	
Participatory data	Dataset is limited to institutional records and does not incorporate participatory or community-generated elements. Efforts are underway to ensure inclusivity by broadening future datasets.	
AI safety and fairness	Known risks include propagation of observational cohort biases into downstream predictive models. Documentation advises caution in clinical translation and outlines appropriate analytic boundaries.	
Inclusion	The sample includes diverse cancer types, both sexes, and a wide age range from 29 to 85 years, but is geographically and institutionally specific. Future data expansions will address representational gaps.	
Annotation platforms and processes	No crowdsourcing or external annotation was used; all data entries were manually curated and verified according to institutional protocol.	
Life cycle documentation	The entire dataset life cycle—from cohort assembly, data curation, laboratory assessment, through archival—is documented using W3C PROV-O provenance and supported by institutional SOPs.	
Record-level metadata	Each record is annotated with demographic, comorbidity, diagnostic, and outcome variables, enabling record-level assessment for fairness, analytic suitability, and audit.	
**Overall FAIR² Badge Compliance**	**Compliant—The dataset qualifies for the FAIR² Badge, meeting all necessary criteria.** Documentation, provenance, standardization, and responsible use guidance support reproducibility, machine learning integration, and ethical reuse. Areas for further enhancement include broader repository indexing and increased demographic diversity in future releases.	

### 3.4 FAIR^2^ certification

## 4 Dataset visualizations

This section provides a visual summary of the demographic, clinical, and molecular characteristics encoded within the dataset of cancer survivors who developed second primary CRC (**Figures 1**–**8**). The figures collectively describe temporal patterns of cancer diagnosis, comorbidity prevalence, treatment exposure, and molecular heterogeneity, emphasizing data structures relevant for downstream analytic workflows and reproducibility. Stratification by first primary cancer type shows variable diagnostic intervals; given the small subgroup sizes, these should be interpreted descriptively.

Age distributions for both first and second primary malignancies demonstrate a rightward shift, with most patients diagnosed with second primary CRC in advanced age groups (**Figure 1**). The temporal interval between primary and subsequent CRC exhibits substantial heterogeneity, particularly for metachronous cases, with intervals ranging from immediate (synchronous) presentation to prolonged latencies exceeding 300 months (**Figure 5**). Stratification by first primary cancer type reveals variable diagnostic intervals, reflecting the diversity in underlying malignancy biology and the surveillance practices represented in the cohort (**Figure 7**).

**Figure 1:**
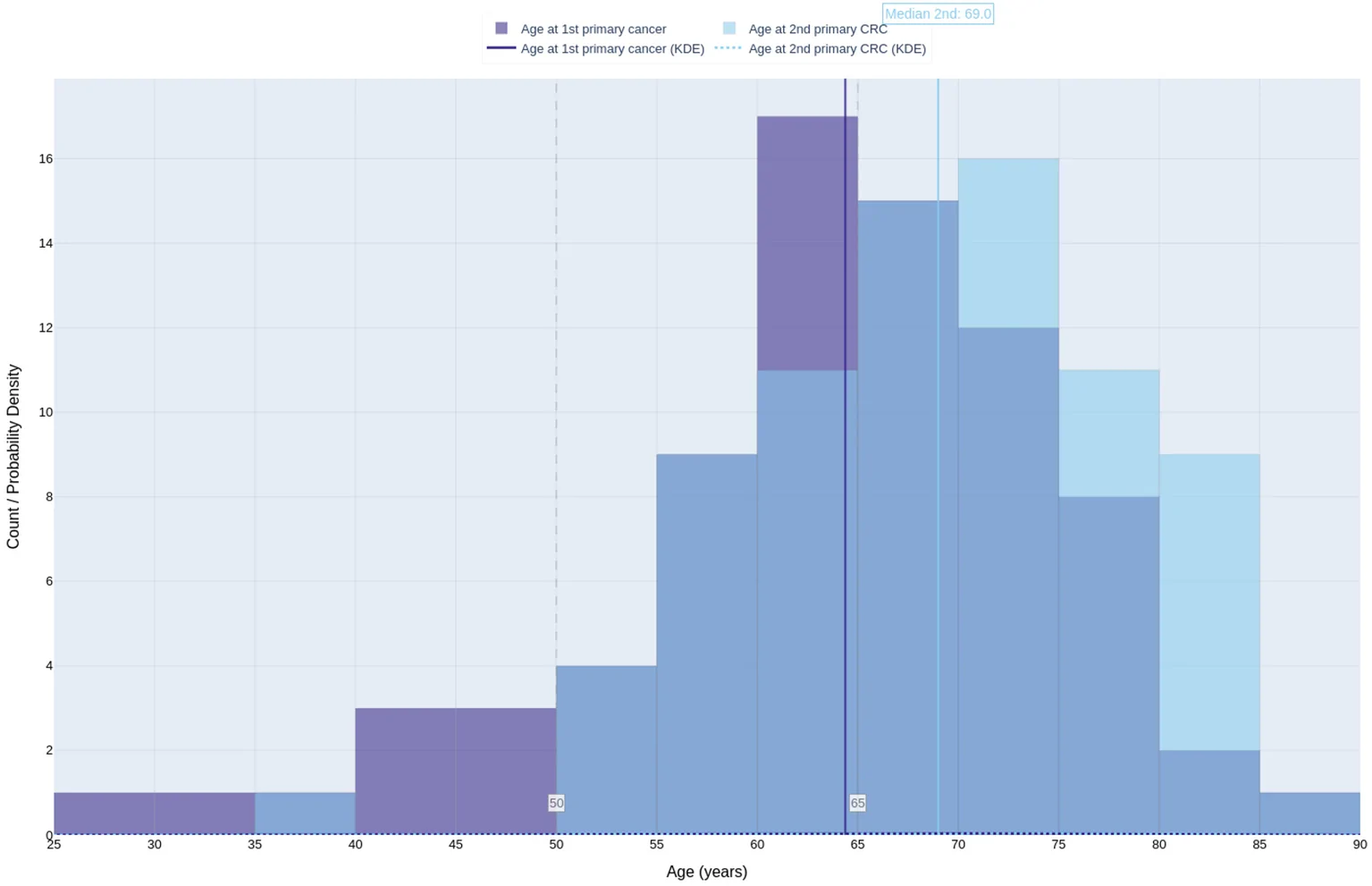
Distribution of patient ages at first and second cancer diagnosis. Age distributions at diagnosis of first primary malignancy and second primary colorectal cancer (CRC) in cancer survivors. Histograms and kernel density estimates (KDEs) reveal a right-shifted age at second CRC diagnosis compared to the first primary cancer. Vertical lines denote group medians, with clinical reference ages (50 and 65 years) marked for context. These patterns highlight common age ranges and latency intervals relevant to CRC risk in cancer survivors. The distribution of patient ages at first primary cancer diagnosis (Age_1) demonstrates a leftward shift relative to the distribution for second primary CRC diagnosis (Age_2), which is displaced toward older age groups. The median age at first cancer diagnosis is 64.4 years, while the median for second primary colorectal cancer is 69.0 years, as indicated by vertical guidelines. Both age distributions exhibit positive skew with prominent peaks between 60 and 75 years. Clinical reference ages of 50 and 65 years are marked, showing that most diagnoses for both primary and secondary cancers occur beyond these thresholds. The kernel density estimates reinforce the histogram findings, with Age_2 consistently displaying greater density in older age intervals. These distributions describe the age structure of this cohort and should not be interpreted as age-specific risk, because the dataset has no reference population.

**Figure 2:**
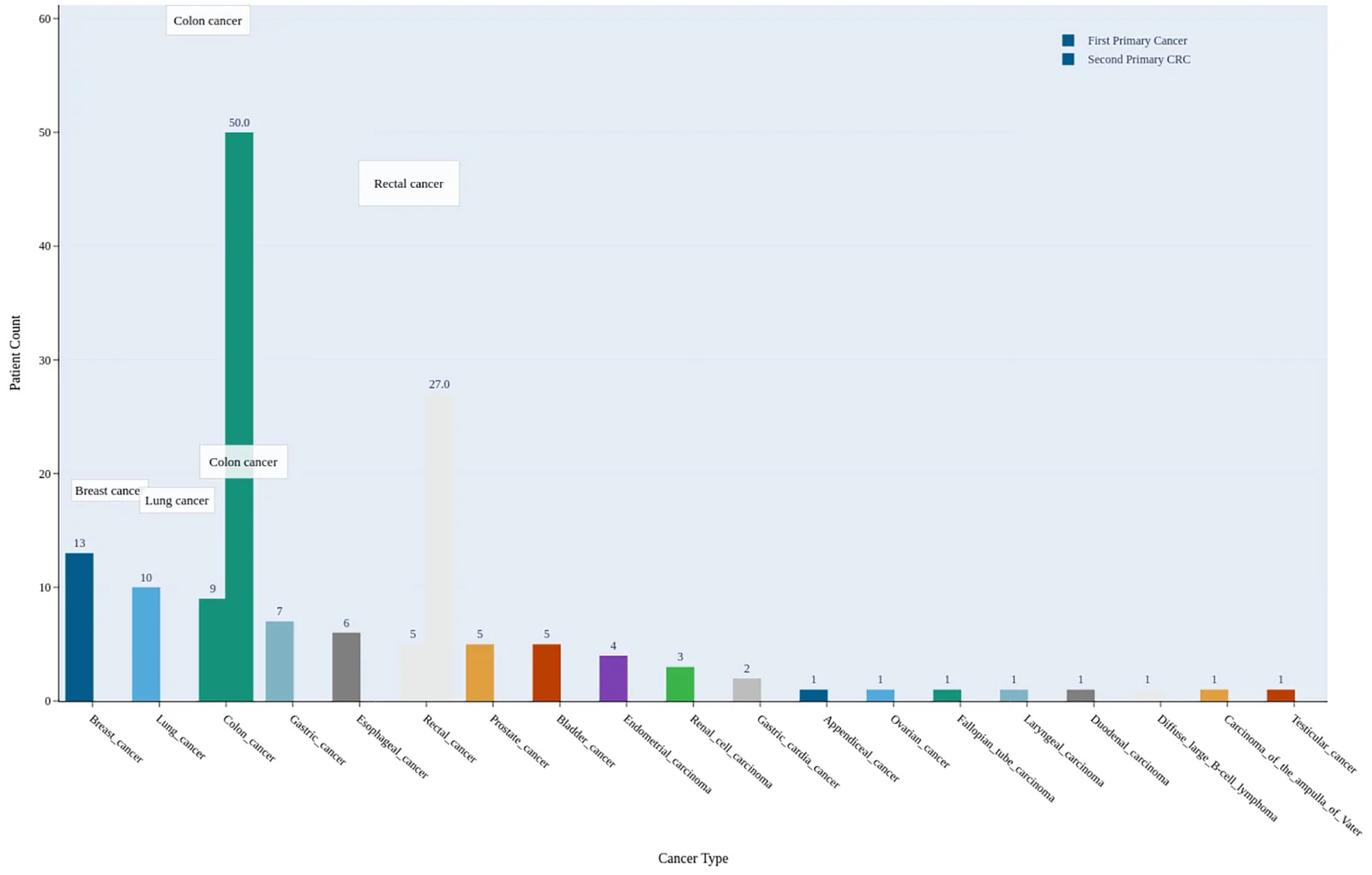
Bar plots of first primary and second primary cancer types. Distribution of cancer types among patients with second primary colorectal cancer, comparing the spectrum of first primary malignancies with the anatomical sites of subsequent colorectal tumors. The most frequent first cancers were breast, lung, and colon, while rectal and sigmoid colon sites predominated among second primary colorectal cancers, highlighting patterns of cancer site diversity and prevalence in this survivor cohort. The distribution of cancer types among patients with second primary colorectal cancer reveals that breast cancer (*n* = 13), lung cancer (*n* = 10), and colon cancer (*n* = 9) are the most common first primary malignancies. For second primary colorectal cancers, colon cancer (*n* = 50) and rectal cancer (*n* = 27) represent the predominant anatomical sites, with colon cancer markedly exceeding other categories in patient count. Other first primary cancer types, including gastric cancer, esophageal cancer, and prostate cancer, appear at substantially lower frequencies. The visualization demonstrates a diverse spectrum of first primary cancer types, while second primary cancers are highly concentrated in colorectal anatomical locations. The top categories in each diagnosis are distinctly labeled, and the overall bar arrangement emphasizes descending prevalence, illustrating clear differences in site-specific cancer incidence between initial primary and subsequent colorectal diagnoses.

**Figure 3:**
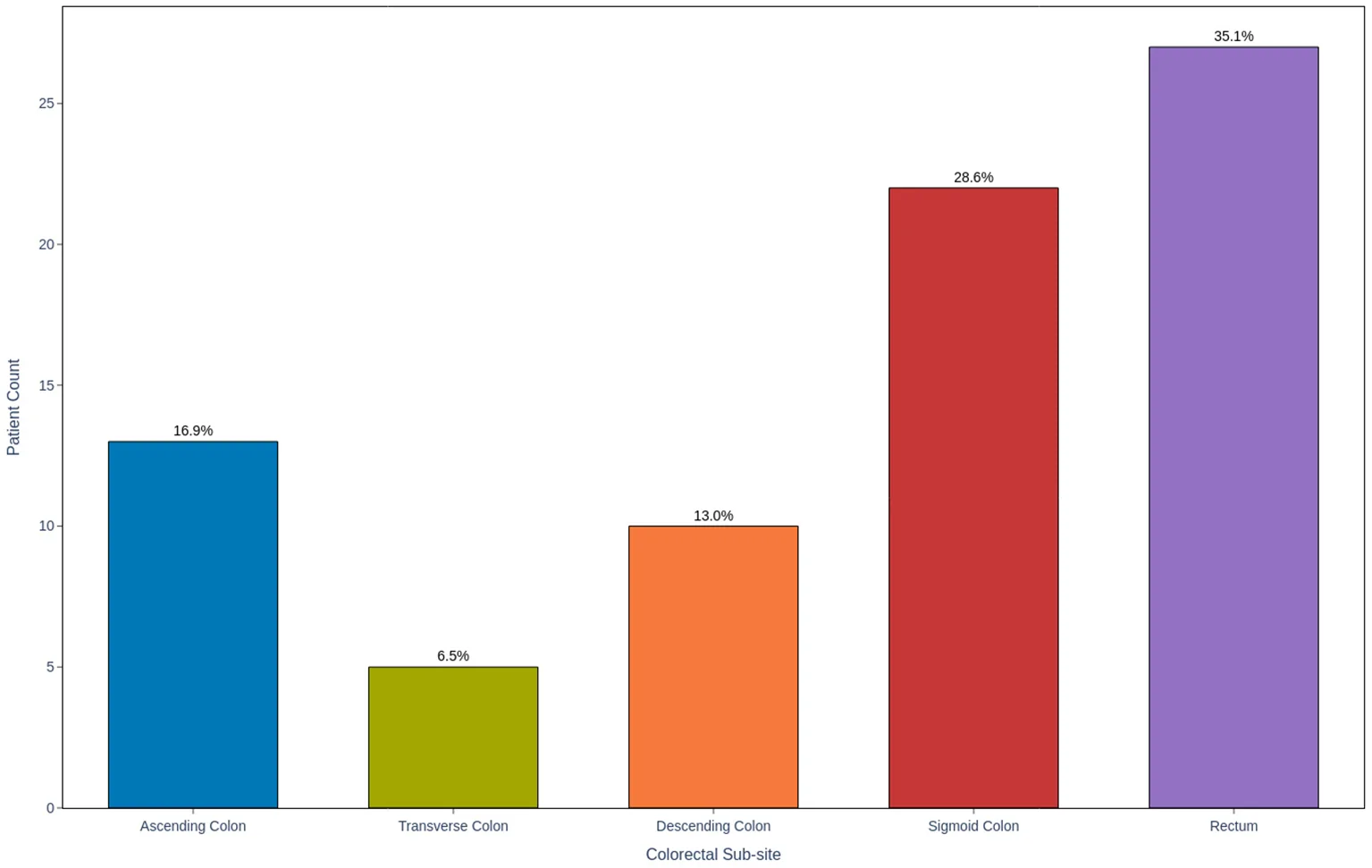
Distribution of anatomical locations for second primary CRC. Distribution of second primary colorectal cancer cases across anatomical sub-sites in cancer survivors, showing patient counts and corresponding percentages. The majority of second cancers are clustered in the rectum and sigmoid colon, with lower frequencies in proximal and transverse segments. The distribution of second primary colorectal cancer cases in cancer survivors is most concentrated in the rectum (35.1%) and sigmoid colon (28.6%), together comprising the majority of cases. Ascending colon (16.9%) and descending colon (13.0%) account for smaller proportions, while the transverse colon (6.5%) has the lowest frequency. This anatomical clustering demonstrates a predominance of distal colorectal involvement in second primary malignancies, with fewer cases observed in proximal and transverse segments.

**Figure 4:**
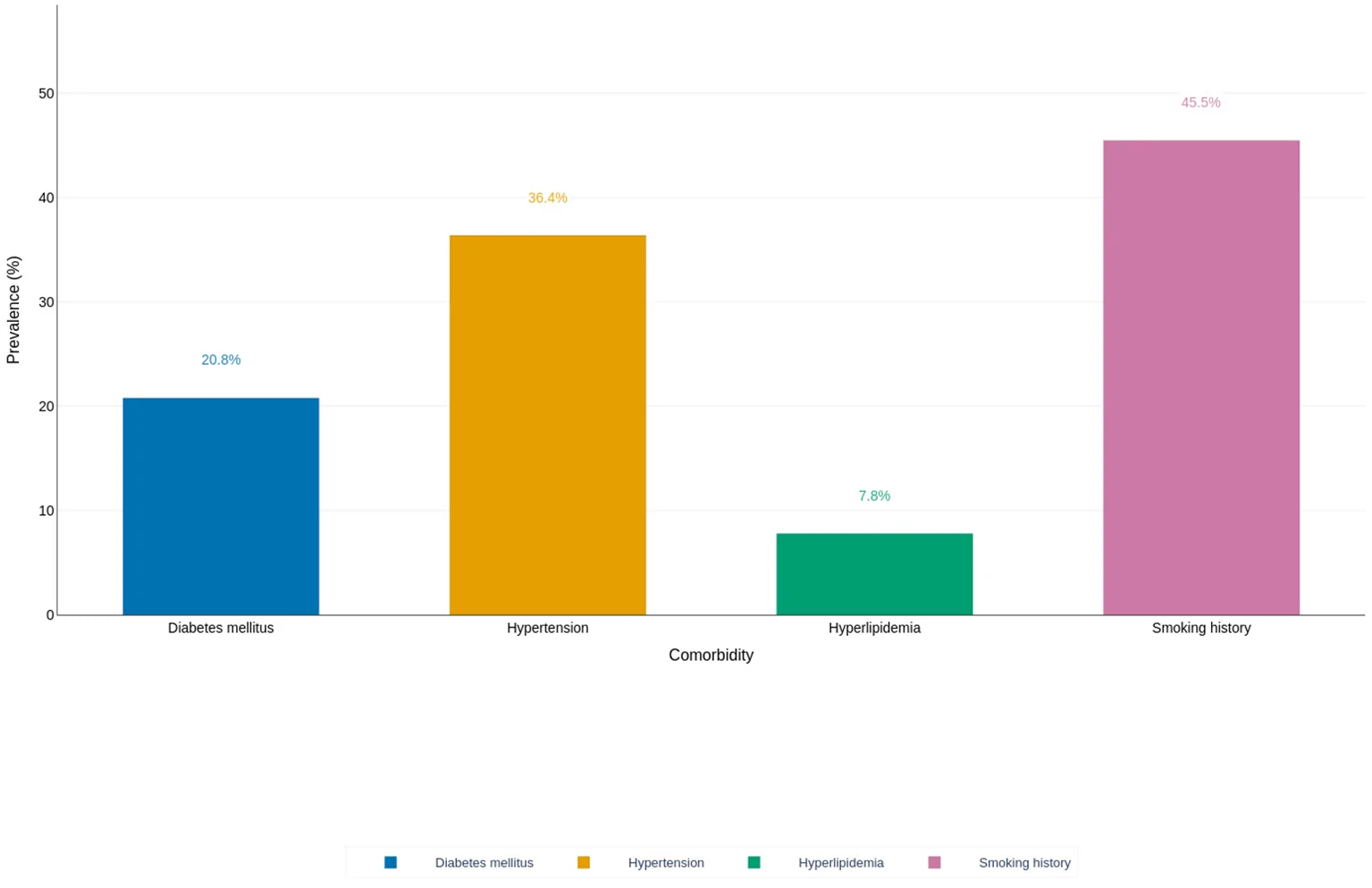
Comorbidity prevalence (diabetes, hypertension, hyperlipidemia, and smoking). Prevalence of diabetes mellitus, hypertension, hyperlipidemia, and smoking history among survivors with second primary colorectal cancer. Hypertension and smoking history are the most common comorbidities in this high-risk cohort, highlighting the substantial cardiometabolic risk profile in cancer survivors. The grouped bar plot demonstrates that among survivors with second primary colorectal cancer, the prevalence of smoking history (45.5%) and hypertension (36.4%) are the highest observed comorbidities. Diabetes mellitus is present in 20.8% of the cohort, while hyperlipidemia is the least prevalent at 7.8%. These data indicate a substantial burden of cardiometabolic and behavioral risk factors, with smoking history and hypertension notably surpassing diabetes and hyperlipidemia in frequency within this high-risk population. Hypertension and smoking history were the most frequently recorded conditions in this cohort. Because there is no comparison group, these proportions cannot be interpreted as an excess risk relative to the general population.

**Figure 5:**
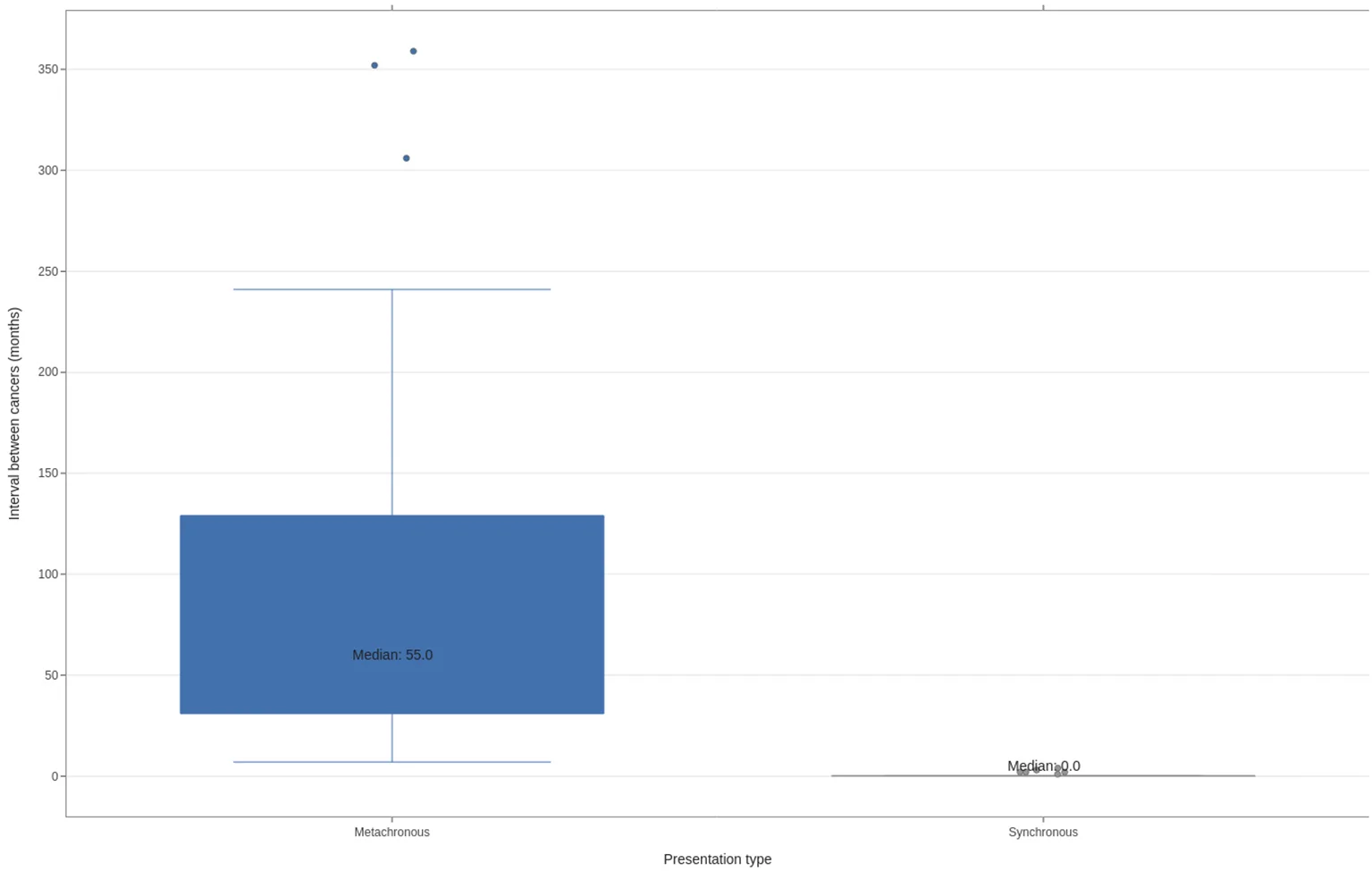
Boxplot of time interval between cancers, stratified by synchronous vs. metachronous presentation. Boxplots depicting the distribution of time intervals (in months) between first and second primary cancers, grouped by presentation type. Metachronous cases (interval > 0) exhibit a wide range of intervals, whereas synchronous cases (interval = 0) cluster at zero months, illustrating the temporal distinction between the two presentation types among the study cohort. Medians are annotated for each group. The boxplot illustrates a clear temporal separation between metachronous and synchronous presentations of second primary colorectal cancer. Metachronous cases (interval > 0 months) display substantial variability in time interval, with a median of 55.0 months and the presence of several high-value outliers exceeding 300 months. The IQR for metachronous cases is broad, indicating heterogeneity in the elapsed time between cancer diagnoses. In contrast, synchronous cases are tightly clustered at an interval of 0 months, with a median of 0.0 months, signifying immediate or simultaneous occurrence of the second primary cancer relative to the first. Outlier points in the metachronous group further accentuate the extended intervals observed for a subset of patients. The plot effectively quantifies and visualizes the temporal distinction between presentation types in the cohort.

**Figure 6:**
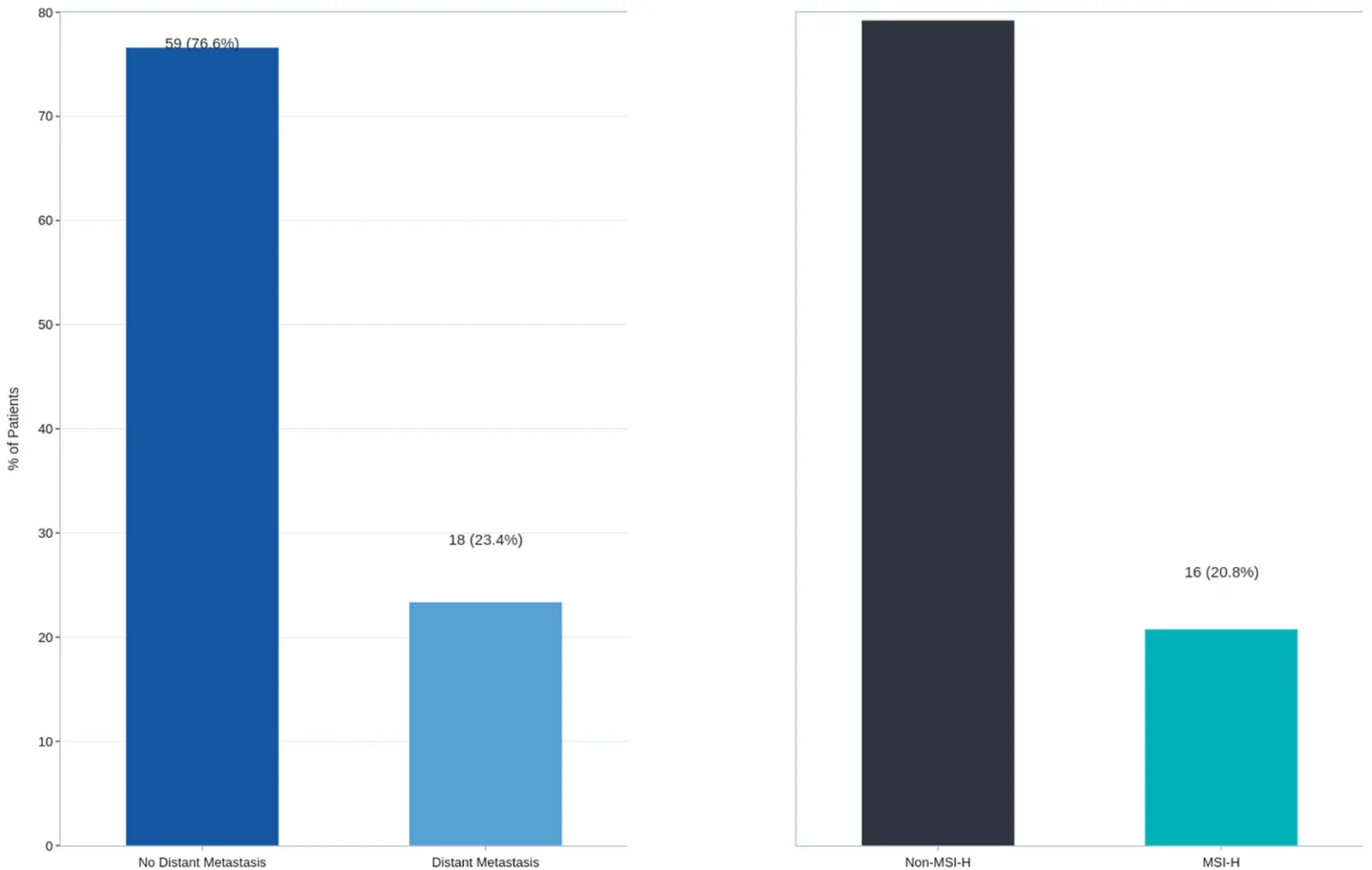
Prevalence of distant metastasis and MSI-H status at CRC diagnosis. Distribution of patients with distant metastasis and MSI-H status at the diagnosis of second primary colorectal cancer (CRC), highlighting the clinical and molecular heterogeneity within the survivor cohort. The visualization demonstrates that, at the diagnosis of second primary CRC in this survivor cohort (*N* = 77), 23.4% of patients (18/77) presented with distant metastasis, while 20.8% (16/77) exhibited MSI-H status. The majority of cases were non-metastatic (76.6%) and microsatellite-stable (79.2%). These proportions indicate a clinically and molecularly diverse population, with substantial frequencies of both advanced disease and MSI-H molecular phenotype among cancer survivors developing second primary CRC.

**Figure 7:**
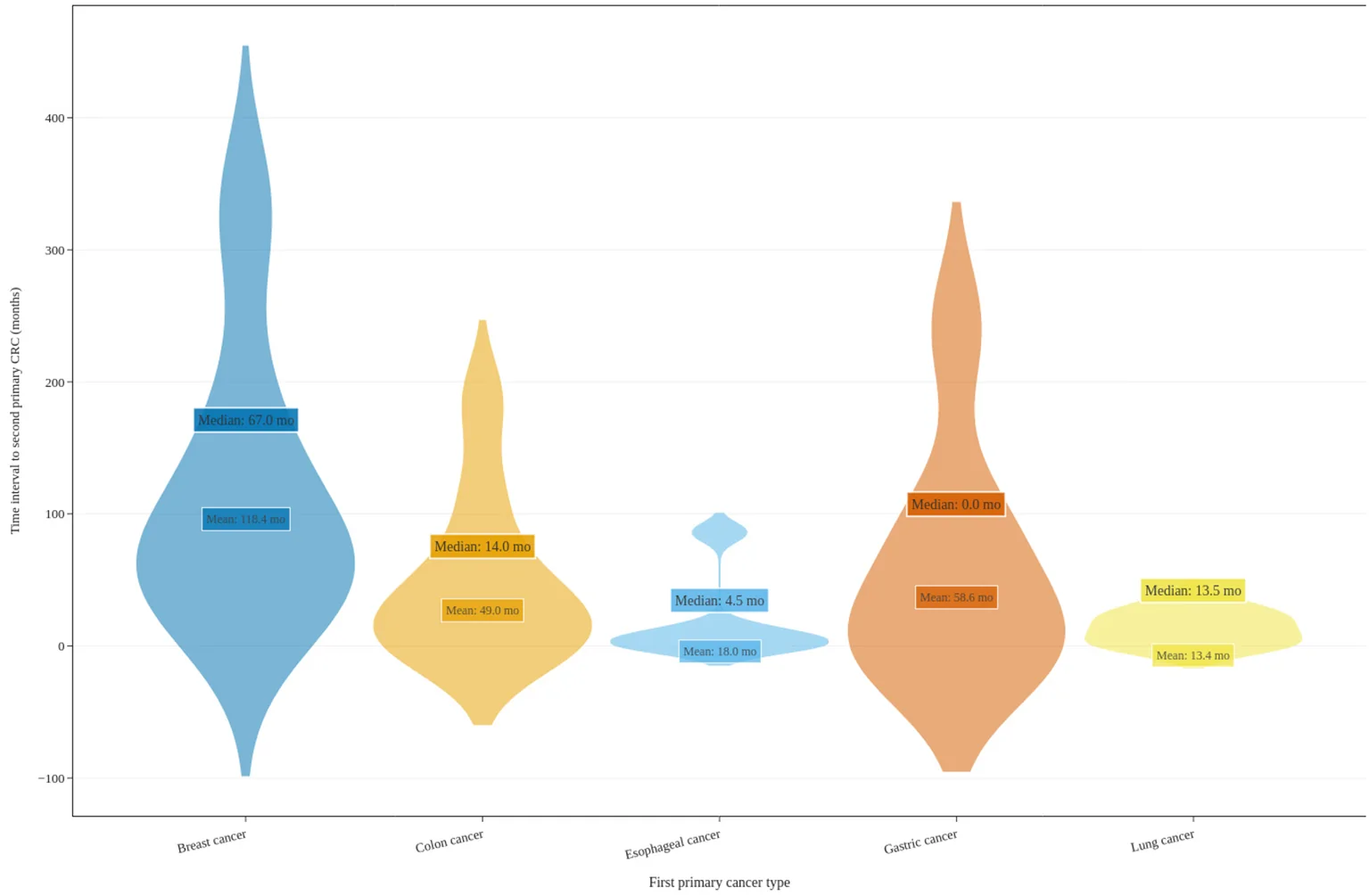
Comparison of time interval between cancers by first primary cancer type. Distribution of time intervals between first primary cancer diagnosis and subsequent second primary colorectal cancer (CRC), stratified by the most common antecedent cancer types. Group medians and means are annotated to highlight potential differences in latency patterns, suggesting varying biological risk or surveillance dynamics by primary cancer type. The interval between the first primary cancer and the second primary CRC varied across the most common first cancer types. Patients with breast cancer as the first malignancy (*n* = 13) had the longest median and mean intervals (median, 67.0 months; mean, 118.4 months), with some intervals exceeding 200 months. Median intervals were shorter for colon cancer (*n* = 9; 14.0 months), lung cancer (*n* = 10; 13.5 months), esophageal cancer (*n* = 6; 4.5 months), and gastric cancer (*n* = 7; 0.0 months). In gastric cancer, the mean (58.6 months) was much higher than the median, reflecting a skewed distribution with several synchronous cases and a few long intervals. These differences are descriptive, are based on very small subgroups, and were not formally tested. They should not be taken as evidence of biological differences in latency; they may instead reflect differences in surveillance after the first cancer (e.g., endoscopic follow-up after gastrointestinal cancers, or staging workup that detects synchronous tumors), the prognosis of the first cancer, referral patterns, and selection bias in a single-institution retrospective cohort.

**Figure 8:**
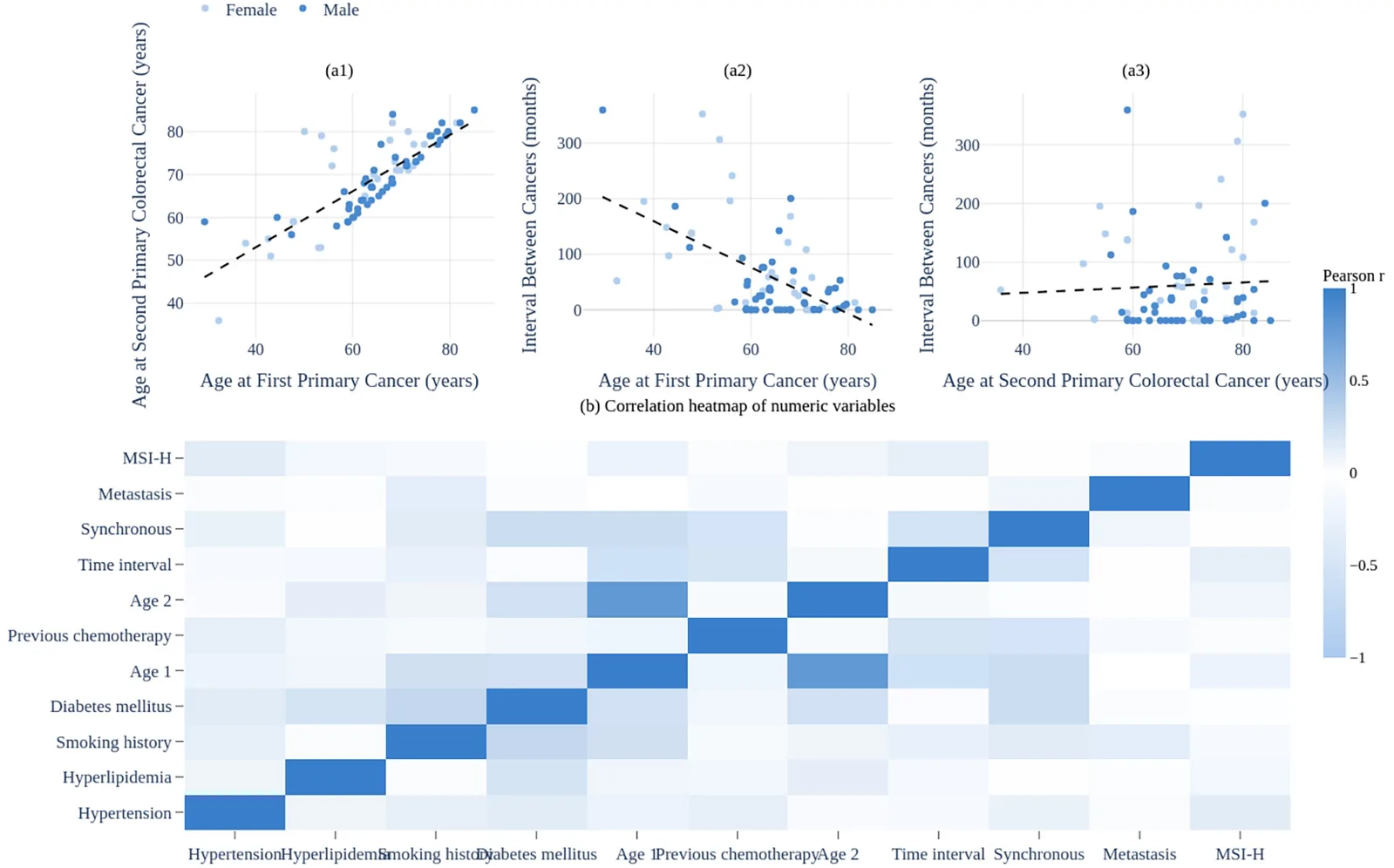
Relationship matrix: scatterplots and correlation heatmap among key continuous variables. Pairwise scatterplots illustrate relationships among age at first cancer, age at second primary colorectal cancer, and diagnostic interval, with points colored by sex and regression lines for trend assessment. The lower panel displays a correlation heatmap for key numeric variables, revealing interdependencies and highlighting distinct timing and demographic patterns within the cohort. The scatterplots demonstrate a strong positive linear association between age at first primary cancer diagnosis and age at second primary colorectal cancer diagnosis, with limited dispersion by sex. In contrast, both age at first cancer and age at second primary colorectal cancer show minimal or weak correlation with the diagnostic interval, as indicated by the flat or negative regression slopes. The correlation heatmap further corroborates these findings, revealing a distinct high positive correlation between age variables, while the time interval is largely independent of age metrics. Most comorbidity and clinicopathological variables exhibit low intercorrelations, with no dominant dependency structure evident among numeric features. These patterns suggest that the interval between malignancies is not primarily determined by patient aging, but instead reflects additional clinical or temporal factors.

Bar plots of antecedent cancer types and anatomical sites of second colorectal malignancy capture the high prevalence of breast, lung, and colon as initial cancers, while second primaries are predominantly localized to the rectum and sigmoid colon (**Figures 2**, **3**). Comorbidity profiles are readily visualized, with hypertension and history of smoking as the leading co-occurring conditions, indicating a substantive burden of cardiometabolic and behavioral risk factors within the population (**Figure 4**).

Visualizations of key clinicopathological variables enable rapid assessment of metastatic disease and MSI-H status at second primary CRC diagnosis, demonstrating both clinical and molecular diversity (**Figure 6**). The scatterplot matrix and corresponding correlation heatmap underscore distinct relationships among age variables and diagnostic interval, illustrating that time to second primary CRC is largely independent of the patient’s age at first diagnosis (**Figure 8**).

Taken together, these graphical summaries delineate the underlying structure and multidimensionality of the dataset and show how the dataset can be used in exploratory statistical analyses and, when pooled with larger cohorts, in modeling studies of second primary malignancies.

## 5 Discussion

### 5.1 Value and applications of the dataset

This dataset provides a structured compilation of clinical, pathological, and comorbidity variables related to patients with second primary CRC after a prior malignancy diagnosis. It details age at diagnosis, cancer site, pathology, metastasis status, and common comorbidities (e.g., hypertension, diabetes, and hyperlipidemia), as well as prior treatment and timing variables. The inclusion of both categorical and numerical variables enables stratification based on demographic, clinical, and disease-specific features, supporting the study of risk patterns, interval timing, and disease course in individuals with sequential malignancies.

The dataset facilitates research into patient trajectories of second primary CRC, including investigation of diagnostic intervals, comorbidity clustering, and treatment histories. Realistic research questions this resource can support include the following: factors associated with the timing between two primary cancers, associations of prior comorbidity or treatment history with metastatic presentation, and the distribution of histopathological subtypes in sequential cancer diagnoses (15). The binary indicators for MSI-H status, metastasis, and synchronous presentation enable subgroup analyses critical for studies focused on genetic and temporal patterns.

Compared to single malignancy registries (16), this dataset addresses a less frequently represented cohort—patients developing CRC subsequent to a different primary malignancy. The granularity of variables, such as the site-specific coding of both first and second cancers, and longitudinal interval measurement, complements more broadly scoped oncology databases by enabling analyses specific to sequential cancer development. The structured, well-annotated fields and machine-readable format facilitate interoperability with population-based databases and support integration in studies utilizing standard terminologies. By describing key clinical and tumor-specific variables alongside time-to-event data, the dataset contributes to research on clinical risk modeling and the epidemiology of multiple primary malignancies.

### 5.2 Limitations of the dataset

Several limitations should be considered when reusing this dataset. First, it includes only 77 patients, with 16 MSI-H cases, from a single institution in Hebei Province, China. This small sample limits statistical power, especially for subgroup analyses (for example, by type of first primary cancer, where most groups include fewer than 15 patients), and limits generalizability to other populations and healthcare settings. The dataset is therefore best suited to descriptive and exploratory analyses, hypothesis generation, and pooled analyses with other cohorts. It is not large enough for the stand-alone development or validation of multivariable prediction or machine-learning models. Any such models would be at high risk of overfitting, should be considered exploratory, and would need external validation in larger multi-institutional cohorts. For the same reason, the multivariable logistic regression described in Section 2 has a low number of events per variable and should be interpreted as exploratory.

Second, the study design, source population, eligibility criteria, and data abstraction procedures are described in Section 2, but the cohort was assembled by complete-case selection. Patients with irretrievable missing or ambiguous data, including unresolved MSI/MMR status, were excluded before the dataset was finalized. As a result, the released dataset has no missing values, but it cannot be used to study the mechanisms of missingness. Excluded patients may differ systematically from included patients (for example, those with incomplete workup, advanced disease, or care partly delivered at other hospitals), which may introduce selection bias. Patients with known hereditary CRC syndromes were also excluded, so the dataset does not represent the full spectrum of MSI-H second primary CRC.

Third, several clinically relevant variables are not available, including TNM stage, tumor grade, detailed treatment information (chemotherapy regimens, radiotherapy, endocrine, targeted, and immune therapies, and surgery for the first and second cancers), recurrence, and survival. The dataset therefore cannot support prognostic or time-to-event analyses. Race, ethnicity, and socioeconomic information were not recorded. Molecular data are limited to MMR/MSI status; other markers such as BRAF V600E, RAS, MLH1 promoter methylation, and germline testing results are not included; thus, sporadic MSI-H tumors cannot be separated from possibly unrecognized hereditary cases.

Fourth, MSI status was mainly inferred from MMR IHC, and PCR was used only when IHC was equivocal. The dataset records the final integrated MSI-H/dMMR classification rather than the separate IHC and PCR results. Several variables (comorbidities, smoking history, prior chemotherapy, and distant metastasis) are binary, without information on dose, duration, or severity (17, 18), and categorical fields such as first cancer type and pathology may combine heterogeneous subtypes. In binary variables, a value of 0 means that the condition was absent; it does not indicate missing data. Finally, the interval between the two cancers may be affected by surveillance intensity, referral patterns, and diagnostic workup after the first cancer, and inter-rater agreement for data abstraction was not formally quantified.

### 5.3 Opportunities for future research

This dataset documents clinical and pathological characteristics of patients diagnosed with both a first primary malignancy and a second primary CRC, accompanied by relevant comorbidity data and treatment histories. The inclusion of variables on synchronous versus metachronous presentation, detailed intervals between diagnoses, and molecular markers such as MSI-H status (19) supports research into temporal patterns, risk factors, and possible mechanisms underlying multiple primary cancers. The standardized variable structure and explicit coding for comorbidities enable harmonization with external cohorts and facilitate reproducible analytic pipelines, especially in studies exploring the interaction between metabolic conditions and malignancy development (20).

Future versions of the dataset could be strengthened by systematic inclusion of additional prognostic variables—such as tumor staging, genetic or genomic markers, and standardized therapeutic regimens—which are relevant for more granular outcome analyses and predictive modeling. Expansion to incorporate longitudinal follow-up, survival outcomes, and recurrence data would further enhance analytical utility, particularly for time-to-event and comparative effectiveness studies. Broader geographic or institutional sampling and linkage to population-based cancer registries could support investigation of environmental or sociodemographic factors. Adhering to established ontologies and integrating richer metadata on variable recording methods and data provenance would advance interoperability and support responsible FAIR²-aligned reuse, including transparent AI model development and external benchmarking against related datasets.

## 6 Conclusions

This dataset provides a rigorously documented record of 77 cases of second primary CRC among cancer survivors, with all core variables systematically mapped to explicit collection and processing procedures. The full data provenance—from initial eligibility determination through variable definition, laboratory evaluation, and data harmonization—is transparently encoded, ensuring users may trace each field to its methodological origin as detailed in the data dictionary and methods description. Uniform ascertainment of key clinicopathological variables, including comorbidities, temporal intervals, prior treatments, cancer types, anatomical location, histopathological subtype, presence of distant metastasis, and MSI-H/MMR status, supports reproducible analysis while minimizing sources of ambiguity.

The dataset structure and accompanying metadata conform to the FAIR² framework, with ontological typing, schema-conformant field definitions, and clearly stated measurement units. Where laboratory parameters such as MSI-H status required multi-step protocols, the workflow is fully specified, including quality control, interpretive criteria, and secondary review or confirmatory testing. Data curation steps—addressing missingness, logical consistency, and cross-field validation—are explicitly documented, ensuring analytic readiness for both traditional statistical approaches and AI/ML applications.

The resource is designed for reuse in descriptive, comparative, and exploratory (hypothesis-generating) studies of second primary colorectal neoplasms in cancer survivors, and for pooling with larger multi-institutional datasets. The dataset’s binary, categorical, and continuous variables enable flexible stratification and support customizable cohort definitions. Its harmonized structure, with machine- and human-readable metadata, facilitates integration with population-level cancer registries or translational research platforms.

By prioritizing detailed provenance, methodological transparency, and strict adherence to ethical and regulatory standards, this dataset maximizes its interoperability, traceability, and relevance for both secondary research and responsible AI workflows. The final archival version, accompanied by structured documentation, is suitable for immediate deployment into analytic pipelines while supporting long-term stewardship and reproducibility within the clinical and computational oncology communities.

## Data Availability

The datasets presented in this study can be found in online repositories. The names of the repository/repositories and accession number(s) can be found below: in the article.
